# Green Synthesis of Nano Zinc Oxide/Nanohydroxyapatite Composites Using Date Palm Pits Extract and Eggshells: Adsorption and Photocatalytic Degradation of Methylene Blue

**DOI:** 10.3390/nano12010049

**Published:** 2021-12-24

**Authors:** Maha S. Elsayed, Inas A. Ahmed, Dina M. D. Bader, Asaad F. Hassan

**Affiliations:** 1 Central Laboratory of Date Palm Research and Development, Agricultural Research Center, Giza 12619, Egypt; 2 Department of Chemistry, Faculty of Science, King Khalid University, Abha 62224, Saudi Arabia; eaahmed@kku.edu.sa (I.A.A.); ddeyaa@kku.edu.sa (D.M.D.B.); 3 Chemistry Department, Faculty of Science, Damanhour University, Damanhour 22511, Egypt; asmz68@sci.dmu.edu.eg

**Keywords:** date palm pits, eggshells, composites, adsorption, photocatalysis, reusability

## Abstract

In this study, zinc oxide nanoparticles (ZnO) and nanohydroxyapatite (NHAP) were prepared in the presence of date palm pits extract (DPPE) and eggshells, respectively. Another four nanocomposites were prepared from ZnO and NHAP in different ratios (ZP13, ZP14, ZP15, and ZP16). DPPE and all nanomaterials were characterized using GC-MS, zeta potentials, particle size distributions, XRD, TEM, EDX, FTIR, and pH_PZC_. The characterization techniques confirmed the good distribution of ZnO nanoparticles on the surface of NHAP in the prepared composites. Particles were found to be in the size range of 42.3–66.1 nm. The DPPE analysis confirmed the presence of various natural chemical compounds which act as capping agents for nanoparticles. All the prepared samples were applied in the adsorption and photocatalytic degradation of methylene blue under different conditions. ZP14 exhibited the maximum adsorption capacity (596.1 mg/g) at pH 8, with 1.8 g/L as the adsorbent dosage, after 24 h of shaking time, and the static adsorption kinetic process followed a PSO kinetic model. The photocatalytic activity of ZP14 reached 91% after 100 min of illumination at a lower MB concentration (20 mg/L), at pH 8, using 1.5 g/L as the photocatalyst dosage, at 25 °C. The photocatalytic degradation of MB obeyed the Langmuir–Hinshelwood first-order kinetic model, and the photocatalyst reusability exhibited a slight loss in activity (~4%) after five cycles of application.

## 1. Introduction

Nowadays, the rapid increase in population and progress in different industries such as textiles, leather, dyeing, cosmetics, paints, and paper manufacturing are causing a major pollution problem in the world [1]. Water is considered as one of the most precious resources for human survival, but it is polluted by several inorganic and organic pollutants such as heavy metal cations, anions, pesticides, antibiotics, dyes, etc. There are about 10,000 different commercial dyes and pigments in existence, and over seven million tons of synthetic dyes are produced annually worldwide [2]. Even at low concentrations, toxic pollutants in water bodies can cause many problems for human and animal health [3,4]. Dyes are classified as anionic, cationic, or neutral, according to their chemical nature [5]. Methylene blue dye is considered to be a dangerous cationic dye [6]. It is a heterocyclic phenothiazine dye with an allowed limit of <0.2 ppm in water according to the EPA (Environmental Protection Agency) [7]. Contamination of water with methylene blue causes many health problems such as mental disorders [8], hypertension, vomiting, nausea, and methemoglobinemia [9].

Approximately 10–15% of the dyes used are released into wastewater, and these dyes and their breakdown products are very toxic for all living organisms; therefore, the removal of dyes from wastewater is important. Many methods are used, such as biological treatment, flotation, photocatalysis, sonocatalysis, catalytic oxidation, coagulation, ultrafiltration, electrochemical methods, and adsorption [10,11]. By comparing the techniques that have been used for the removal of pollutants, we found that adsorption and photocatalysis techniques are considered to be the most important techniques for wastewater purification, because both are economically efficient and fast, with reusable solid material and low energy use. Others techniques are expensive, potentially hazardous to the environment and living organisms, and not simple, leading to residual toxic species [12]. Photocatalysts, as semiconductors, are very effective in organic pollutant degradation and are characterized by simple reusability, simple processes, and a higher percentage degradation efficiency, especially at lower concentrations of pollutant. Many photocatalysts are used, such as TiO_2_, CdS, ZnS, WO_3_, ZnO, etc. In some photocatalytic processes, the catalyst activity can be enhanced via chemical modifications such as composite formation and heavy metal decoration, where surface modification can enhance the band gap of the photocatalysts. Composite formation is characterized by spreading of the photocatalyst particles in the newly formed composite matrix and enhancement of reusability functions.

Nanoparticles are good adsorbents with unique physicochemical and biological properties over their bulk phase, but it is difficult to remove them from solution due to their fine particle size. This problem can be solved by forming stable composite samples [13]. Among these nanoparticles, nanohydroxyapatite, which has the molecular formula Ca_10_(PO_4_)_6_(OH)_2_, is used in medical and environmental applications due to its bioactivity, porous structure, and functional groups on the surface [14,15,16]. Nanohydroxyapatite is produced either by chemical processes or by calcination of bio-wastes such as fish bones, bovine bones, pig bones, eggshells, or sea-scallop shells. The production of nanohydroxyapatite from natural sources such as eggshells for environmental applications is an accepted solution for reusing waste eggshells and a low-cost method of nanomaterial production [17]. Green synthesis of metal oxide nanoparticles using plant extracts is a promising alternative to traditional methods of chemical synthesis. In this study, we use extract of date palm pits in the preparation of nano zinc oxide, where the date palm pits extract works as a capping agent and prevents nano zinc oxide coagulation. The pits account for around 10–15% of the total date fruit weight. There are more than 100 million date palm trees worldwide [18], spread over all the hot regions, which makes the use of date palm pits as a raw material source for industrial purposes a promising project.

In this report, we discuss the preparation of nano zinc oxide and nanohydroxyapatite composite samples with different ratios of nanohydroxyapatite to nano zinc oxide. Various physicochemical tools were used in the characterization of the prepared materials, such as GC-MS, pH_PZC_, zeta potentials, FTIR, XRD, TEM, DLS, and EDX. The adsorption of MB via the static adsorption method using the prepared solid adsorbents is discussed. The photocatalytic degradation of methylene blue was investigated using the prepared photocatalysts under different application conditions.

## 2. Materials and Methods

### 2.1. Materials

Date palm pits and eggshells were collected from a local market and restaurant in Cairo, Egypt. The date palm pits and eggshells were washed to remove any impurities, dried at 105 °C, and ground into fine particles. Zinc nitrate and methylene blue were purchased from Sigma-Aldrich Co., St. Louis, MO, USA. Diammonium hydrogen phosphate and ammonium hydroxide were purchased from El-Nasr Pharmaceutical Chemicals Co., Cairo, Egypt. All chemicals were used without further purification.

### 2.2. Methods

#### 2.2.1. Preparation of Date Palm Pits Extract

The extract of date palm pits was prepared by placing 10 g of ground pits in 500 mL of distilled water, followed by stirring by magnetic stirrer for 24 h. After that, the extract was filtered. The residual powder was found to weigh 6 g, indicating that the obtained extract contained about 4 g of the dissolved date palm pits substances (DPPE).

#### 2.2.2. Biosynthesis of Nano Zinc Oxide and Composite Samples

To prepare nano zinc oxide, 1 g of zinc nitrate was dissolved in 50 mL of date pits extract, placed in a 250 mL beaker, and kept under mechanical stirring at 240 rpm for 2 h. After complete homogenization, a few drops of NH_4_OH were added dropwise till precipitation occurred. Then, the precipitate was centrifuged at 6000 rpm, washed with distilled water, dried at 110 °C, and finally calcined at 550 °C for 3 h (ZnO). 

Nanohydroxyapatite was obtained from eggshell powder as reported previously, with slight modification [19]. The obtained eggshell powder was heated to 800 °C for 4 h (heating rate of 10 °C/min) to convert all the calcium carbonate into calcium oxide and to completely decompose all organic compounds [20]. The CaO was ground, and 5.0 g of the powder was mixed with HNO_3_ (1.5 M) under magnetic stirring. Diammonium hydrogen phosphate (0.5 M) was added dropwise to the solution under constant stirring at room temperature. The stoichiometric Ca/P ratio was 1.67, and the reaction mixture was kept at pH 10–11 overnight using NH_4_OH. Then, the formed precipitate was filtered, washed with deionized water several times to remove any excess reagent, and dried at 110 °C overnight. The dried precipitate was calcined at 900 °C for 3 h to obtain nanohydroxyapatite. The sample was sieved to give a mean particle diameter of 1.8–2.0 mm (NHAP) and stored. The following equations represent the preparation steps:

CaCO_3_ → CaO + CO_2_ (1)

CaO + 2HNO_3_ → Ca(NO_3_)_2_ + H_2_O (2)

10Ca(NO_3_)_2_ + 6(NH_4_)_2_HPO_4_ + 8NH_4_OH → (Ca)_10_(PO_4_)_6_(OH)_2_ + 20NH_4_(NO_3_) + 6H_2_O (3)

To prepare nano zinc oxide/nanohydroxyapatite composite samples, 1.5 g of nanohydroxyapatite was dispersed in 50 mL of date palm pits extract and sonicated for 2 h until homogenization, followed by the addition of a certain weight of zinc nitrate to give various ZnO:NHAP ratios (1:3, 1:4, 1:5, and 1:6) and the dropwise addition of NH_4_OH until complete precipitation occurred. The obtained precipitates were treated as in the case of nano zinc oxide preparation (ZP13, ZP14, ZP15, and ZP16). 

### 2.3. Characterization of Date Palm Pits Extract and Prepared Nano Solid Samples

Various physicochemical techniques were used for the characterization of the date palm pits extract and prepared solid nanomaterials.

#### 2.3.1. Characterization of Date Palm Pits Extract

Date palm pits extract (DPPE) was characterized using gas chromatography–mass spectrometry analysis (GC-MS). The system (Agilent Technologies, Santa Clara, CA, USA) was equipped with a gas chromatograph (7890B) and a mass spectrometer detector (5977A). The GC was equipped with an HP-5MS column (30 m × 0.25 mm internal diameter and 0.25 μm film thickness). Analysis was carried out using hydrogen as the carrier gas at a flow rate of 1.0 mL/min with a splitless sample of 2.0 µL of the extract as the injection volume, and the temperature program was 50 °C/min followed by 10 °C/min up to 300 °C for 20 min. The injector and the detector were held at 250 °C. Mass spectra were obtained by electron ionization (EI) at 70 eV, using a spectral range 30–700 as *m*/*z* and a solvent delay of 9 min. The mass temperature was 230 °C (source) and 150 °C (quad). Identification of the different constituents was determined by comparing the spectrum fragmentation patterns with those stored in the Wiley and NIST mass spectral libraries.

#### 2.3.2. Characterization of the Prepared Nano Solid Samples

The zeta potentials of the NHAP, ZnO, ZP13, ZP14, ZP15, and ZP16 samples and the particle size distributions were determined using a Zetasizer Nano (NanoZS, Malvern, UK). X-ray diffraction patterns (XRD) were investigated for ZnO, ZP13, ZP14, ZP15, and ZP16 using a Bruker AXS D8 Advance diffractometer (Karlsruhe, Germany). Fourier transform infrared spectroscopy (Mattson 5000 FTIR spectrometer, San Mateo, CA, USA) was performed for NHAP, ZnO, ZP13, and ZP16 in the range 400–4000 cm^−1^ using a Mattson 5000 FTIR spectrometer to investigate the surface chemical functional groups. Points of zero charges (pH_PZC_) for NHAP, ZnO, ZP13, ZP14, ZP15, and ZP16 were studied by mixing 50 mL of 0.15 M NaCl solution in closed bottles, then controlling the pH in the range from 2–12 using 0.05 M HCl and/or NaOH. The bottles were shaken for about 72 h, and the values of pHfinal were evaluated using a Hanna pH meter (San Mateo, CA, USA), where pH_PZC_ is defined as the point at which pH_final_ equals pH_initial_ [21]. Prior to pH_PZC_ determination, the solubility of the ZnO nanoparticles was investigated in media with different pH values (pH of 1–12 using NaOH and/or HCl), where we observed that the nanoparticles were insoluble under the effect of different pH values. This may be related to the unique characteristics of nanomaterials. TEM images of NHAP, ZnO, ZP13, ZP14, ZP15, and ZP16 solid nanomaterials were studied using an FEI Tecnai G20 Super twin, double tilt instrument (Eindhoven, The Netherlands), while EDX analyses of the samples were carried out using an (Oxford Instruments Link Isis system, Abingdon, UK).

### 2.4. Adsorption and Photocatalytic Degradation of Methylene Blue

#### 2.4.1. Adsorption of MB onto the Prepared Solid Adsorbent

The effect of adsorbent dosage for ZP13 and ZP14, as two selected solid adsorbents, was investigated at 25 °C by mixing 0.5–3.0 g/L of adsorbent with 50 mL of 200 mg/L MB solution in a 250 mL Erlenmeyer flask, at pH 7, shaking for 24 h, and filtering. Then, the concentration of methylene blue in the filtrate was measured at a λ_max_ of 664 nm using an HPST UV/vis spectrophotometer (HPST, S.R.O.Provozovna., Slechtitelu, Czech Republic). The removal percentage was calculated according to the following equation:

(4) $$R\%=\frac{(C_{o}-C_{e})\;100}{C_{o}}\;$$

where *C_o_* and *C_e_* (mg/L) are the concentrations of MB at the beginning and at equilibrium, respectively. 

The effect of the initial pH of the methylene blue solution was investigated for the adsorption onto ZP13 and ZP14 in the pH range of 1–12, using 50 mL of 200 mg/L MB solution at 25 °C, after 24 h of shaking time using 0.05 g of solid adsorbents. 

The effect of shaking time was determined by mixing 1.8 g/L of adsorbents (ZP13 and ZP14) with 100 mL of 200 mg/L MB aqueous solution. After certain time intervals (up to 70 h), the concentration of MB was determined in the solution. The adsorption capacity of the adsorbent (*q_t_*, mg/g) at time *t* was calculated as follows:

(5) $$q_{t}=\frac{(C_{o}-\;C_{t})\;V}{W}\;$$

Here, *C_t_* (mg/L) is the liquid-phase concentration of MB at time *t* and *W* (g) is the mass of solid adsorbents. The variation of dye adsorption with time was determined according to pseudo-first-order and pseudo-second-order kinetic models, as shown in Equations (6) and (7), respectively.

(6) $$\log\left(q_{e}-q_{t}\right)=logq_{e}-\frac{k_{1}}{2.303}\;t\;\;\;\;\;\;\;$$

(7) $$\frac{t}{q_{t}}=\frac{1}{k_{2}q_{e}{}^{2}}+\frac{t}{q_{e}}$$

where *k*_1_ (min^−1^) and *k*_2_ (g/mg min) are the pseudo-first-order and pseudo-second-order rate constants, respectively. 

The effect of the initial MB concentration was investigated for NHAP, ZnO, ZP13, ZP14, ZP15, and ZP16 in the range 10–800 mg/L, with 1.8 g/L as the adsorbent dosage, at pH 7, and after a shaking time of 24 h. The linear Langmuir adsorption equation was applied, and the maximum adsorption capacity (*q_m_*, mg/g) was determined, where *q_e_* (mg/g) is the equilibrium adsorption capacity. Equation (8) represents the linear Langmuir adsorption process, where *b* (L/mg) is the Langmuir constant.

(8) $$\frac{C_{e}}{q_{e}}=\frac{1}{b\;q_{m}}+\frac{C_{e}}{q_{e}}$$

A dimensionless separation factor (*R_L_*) was used to investigate whether the adsorption process was favorable, unfavorable, or irreversible. The dimensionless separation factor can be calculated from the following equation:

(9) $$R_{L}=\frac{1}{1+b\;C_{o}}$$

The adsorption process is irreversible in the case where *R_L_* = 0, unfavorable when *R_L_* > 1, and favorable if 0 < *R_L_* < 1 [22].

#### 2.4.2. Photocatalytic Degradation of MB Using the Prepared Solid Nanocatalyst

Photocatalytic degradation of MB was performed using a Pyrex reactor with length of 10 cm and width of 20 cm, containing 200 mL of 20 mg/L MB solution and 0.2 g of catalyst. The reactor was illuminated with a TUV TL lamp with energy of 8 W, a 16 mm diameter, and a slim double-ended UV-C of 253.7 nm. The degradation was determined by the removal of 2 mL of solution at different time intervals, and the residual MB concentration was determined as described above. The effect of photocatalyst dosage (0.25–2.00 g/L) was examined for ZP14 using 200 mL of 20 mg/L MB at 25 °C with 100 min of illumination. The effect of pH on the photocatalytic efficiencies for ZnO, ZP13, and ZP14 was studied for the pH range 1–12, for MB degradation under UV illumination using 200 mL of 20 mg/L MB solution and a 2 g/L catalyst dose, after 100 min and at 25 °C.

The photocatalytic activities of the prepared materials were investigated with 100 min of illumination at pH 7, with 2.0 g/L as the catalyst dosage and at 25 °C, while the photolysis was studied in the absence of any photocatalysts. Prior to the photocatalysis studies, the mixtures were kept in the dark for 30 min in the absence of any radiation, to examine the adsorption process as a blank experiment.

A kinetic model was applied for investigating the photocatalytic degradation of methylene blue in the presence of ZnO, ZP13, ZP14, ZP15, and ZP16 by applying the Langmuir–Hinshelwood first-order kinetic behavior (Equation (10)).

(10) $$Log\frac{C_{o}}{C_{t}}=\frac{Kkt}{2.303}=\frac{K_{app}}{2.303}\;t$$

Here, *C_o_* (mg/L) is the initial concentration of MB, *C_t_* (mg/L) is the residual concentration of MB after time *t*, *k* is the reaction rate constant, and *K* is the MB adsorption coefficient (L/mg). *K_app_* is the apparent first-order rate constant.

ZP14 was selected for investigating the nanocatalyst reusability. The ZP14 reusability was determined after five cycles of the MB degradation process. The catalyst was filtered after every experiment, gently washed with double-distilled water and dried at 60 °C before use in the next cycle.

## 3. Results and Discussion

### 3.1. Characterization of Date Palm Pits Extract and Prepared Solid Materials

#### 3.1.1. Chemical Characterization of Date Palm Pits Extract

**Figure 1:**
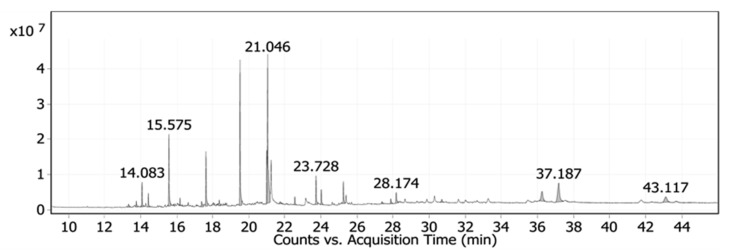
GC-MS chromatogram of DPPE showing the peaks for various components.

Date palm pits extract (DPPE) was explored for various possible components using GC-MS. Twenty-four compounds were identified and the active principles, molecular formulas (MF), concentrations (peak area %), and retention times (RT) are shown in Figure 1 and Table S1. The major compounds detected in the GC-MS analysis of date pits extract were 9-octadecenoic acid, (E), TMS derivative (23.81%), palmitic acid, TMS derivative (16.12%), trans-13-octadecenoic acid (8.72%), and dodecanoic acid trimethylsilyl ester (8.46%). 

DPPE plays an important role as a stabilizing agent and inhibits nanoparticle agglomeration due to the presence of various active chemical compounds such as phenols, polysaccharides, and other materials that are rich in hydroxyl groups [23]. Negatively polar oxygen atoms of the hydroxyl chemical functional groups in DPPE are able to connect with ZnO via the ionic–dipolar interaction to prevent nanoparticle aggregations, in addition to the electrostatic interactions that reduce the activity of divalent metal ions and cause nucleation in the polymer matrix [24]. Thus, the molecular chains of the natural polymer extract prevent the aggregation and growth of the produced ZnO nanoparticles, thereby leading to the formation of nanostructures in the reaction medium [25].

#### 3.1.2. Characterization of the Prepared Solid Materials

**Figure 2:**
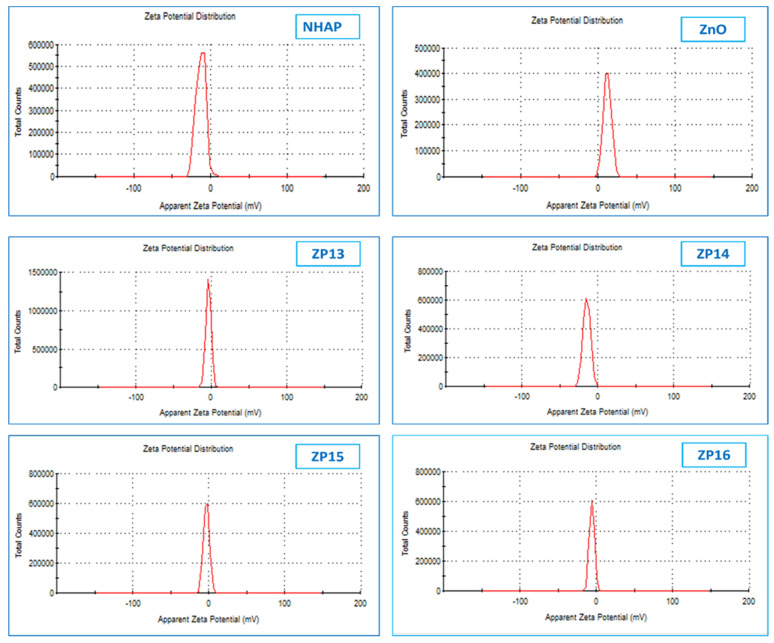
Zeta-potential analysis of synthesized NHAP, ZnO, ZP13, ZP14, ZP15, and ZP16.

Zeta potential studies were used to identify the surface charges obtained by all the prepared nanoparticles, where the values of the zeta potentials confirm the samples’ stability [26]. It is known that particles will repel each other and prevent aggregation of nanoparticles if the particles in suspension have large negative or positive zeta potential values. The zeta potentials of the prepared nanosolid samples are displayed in Figure 2. The zeta potentials for NHAP and ZnO were found to be −12.5 and +12.2 mV, respectively, while in case of all the other prepared composite samples (ZP13, ZP14, ZP15, and ZP16) the zeta potentials were in the negative range of 2.52–4.8 mV. The values of the negative zeta potentials of the composites increased with an increase in the amount of NHAP. The previous observations confirm the successful framework of the formed composites between the two components (NHAP and ZnO).

**Figure 3:**
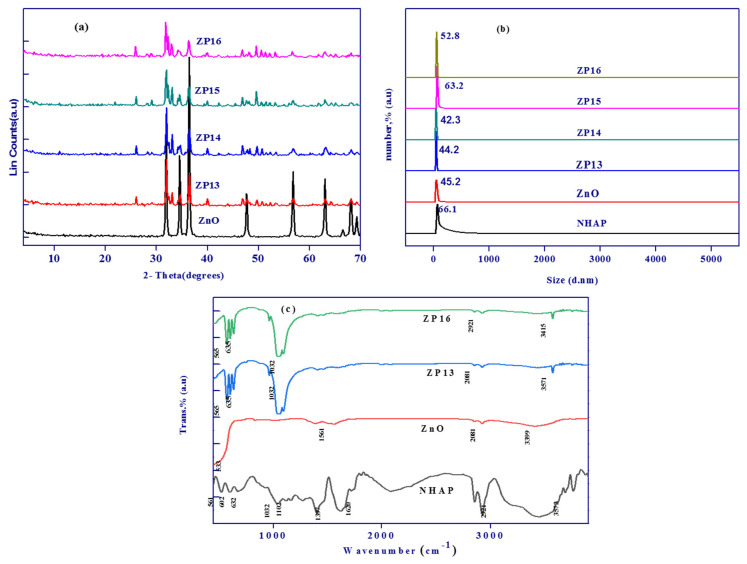
XRD results for ZnO, ZP13, ZP14, ZP15, and ZP16 (**a**); particle size distribution of all the prepared solid materials (**b**); FTIR spectra of NHAP, ZnO, ZP13, and ZP16 (**c**).

X-ray diffraction patterns for ZnO, ZP13, ZP14, ZP15, and ZP16 are displayed in Figure 3a. The nano zinc oxide sample shows sharp peaks located at 2*θ* values of 31.7, 34.4, 36.2, 47.5, 56.6, and 62.8°, corresponding to the lattice planes of (100), (002), (101), (102), (110), and (103), respectively, based on JCPDS card no. 01-073-8417. The observed sharp peaks indicate the crystalline hexagonal wurtzite structure of nano zinc oxide, as reported in many published studies [27,28]. The composite samples (ZP13, ZP14, ZP15, and ZP16) that were prepared from nano zinc oxide and nanohydroxyapatite exhibited peaks related to nanohydroxyapatite at 2*θ* values of 25.8, 31.9, 32.2, 34.1, 39.8, and 49.5°, corresponding to the diffraction planes (002), (211), (112), (202), (130), and (213), respectively, proving that the composites are affected by the textural structure of NHAP more than that of nano zinc oxide [29,30]. The calculated particle sizes from the XRD data for ZnO, ZP13, ZP14, ZP15, and ZP16 were found to be 35.1, 40.2, 38.4, 51.2, and 42.8 nm, respectively.

Dynamic light scattering is broadly a technique used for the determination of solid particle size in colloidal solutions (Figure 3b). The calculated average particle sizes of the prepared ZnO, NHAP, ZP13, ZP14, ZP15, and ZP16 samples in the colloidal phase were found to be in the range of 42.3–66.1 nm, in accordance with those measured using the XRD technique. It was clear that the solution containing zinc oxide nanoparticles had an average particle size of 45.2 nm, which indicated that the obtained nano zinc oxide particles were monodispersed in nature, and that DPPE had an effective character as a capping agent in the synthesis of nano zinc oxide particles [31]. The particle size for NHAP was more than that determined for ZnO by about 1.46 times, when it was prepared in the absence of any capping agent. The formed composites exhibited higher particle sizes with an increase in the amount of NHAP, as observed in the case of ZP15 and ZP16 [32].

Figure 3c exhibits the FTIR spectra of NHAP, ZnO, ZP13, and ZP16 to identify the surface chemical functional groups. The FTIR curve for the nanohydroxyapatite particles showed absorption peaks at 602 and 561 cm^−1^, corresponding to the PO_4_^3−^ triply degenerate bending modes of the O–P–O vibrations, while the peaks at 1032 and 1102 cm^−1^ are related to the triply degenerate asymmetric stretching vibrations of P–O bonds [33]. The peaks present at 3572 and 632 cm^−1^ correspond to vibration of the apatite –OH group [34]. In the case of zinc oxide nanoparticles, the characteristic peaks located at 533 and 3572 cm^−1^ are related to Zn–O bond and –OH stretching vibrations, while bands located at 1561 and 2912 cm^−1^ may be related to C=O stretching in polyphenols and/or C=C stretching in the aromatic ring and alkane C–H stretching of the residual organic extract groups used as a capping agent [35]. ZP13 and ZP16 exhibited the same FTIR spectra with a slight decrease in transmittance intensity. The spectra of the ZnO/NHAP composites showed the characteristic peaks of both ZnO and NHAP, especially at 565, 635, 1032, 2081, and 3571 cm^−1^ which confirms the formation of the composites.

The importance of the point of zero charge measurement for solid adsorbents is related to the fact that at pH > pH_pzc_, the surface of a solid acquires a net negative charge, while it is positive at pH < pH_pzc_. The measured pH_PZC_ values for NHAP, ZnO, ZP13, ZP14, ZP15, and ZP16 were found to be 7.4, 8.4, 8.1, 8.0, 7.8, and 7.6, respectively. The slight observed decreases in the points of zero charge of the composites are directly related to the increase in NHAP content [36].

**Figure 4:**
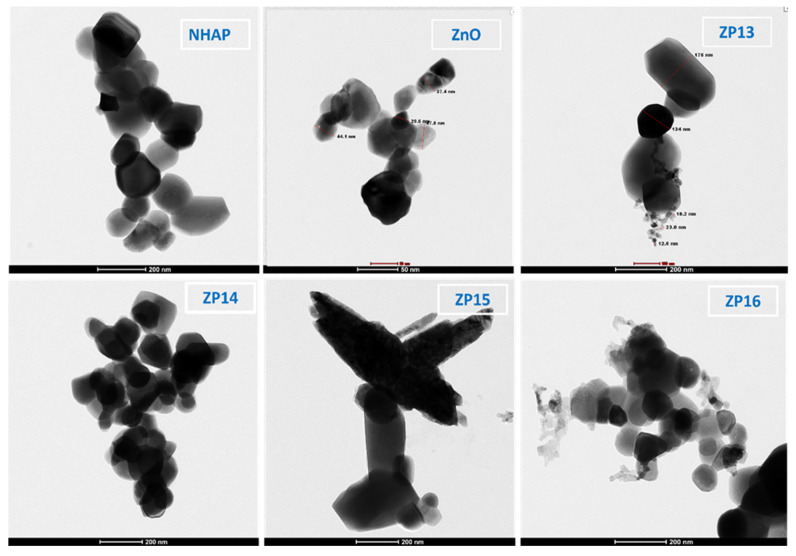
TEM images of NHAP, ZnO, and different ZnO/NAHP composite samples.

TEM micrographs for ZnO, NHAP, ZP13, ZP14, ZP15, and ZP16, are shown in Figure 4; they are in agreement with the dynamic light scattering and XRD particle size determinations. TEM showed nano-sized particles with an average crystal size of around 46.2 nm for all the prepared solid materials. Nanohydroxyapatite, nano zinc oxide, and composites with lower percentages of NHAP showed a spherical shape, while composites with higher NHAP contents (ZP15 and ZP16) were more amorphous with an aggregated shape. 

Energy-dispersive X-ray analysis was used for the elemental analysis of all the prepared samples, as shown in Figure S1. In the case of the NHAP sample, the strong peaks observed in the spectrum are related to calcium, phosphate, and oxygen. Zinc and oxygen peaks were observed in the case of the ZnO nanoparticle sample, which confirms the high purity of the prepared solid nanoparticle samples. For all the composite samples, we found that four peaks appeared, corresponding to calcium, phosphate, zinc, and oxygen. In addition, the appearance of these peaks confirmed the formation of the composite nanoparticles for ZP13, ZP14, ZP15, and ZP16 [37].

### 3.2. Adsorption of Methylene Blue

#### 3.2.1. Effect of Dosage

**Figure 5:**
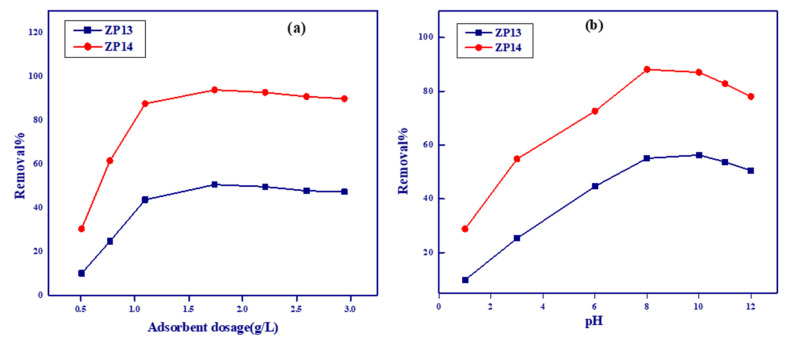
Effect of adsorbent dosage (**a**) and effect of pH (**b**) on adsorption of methylene blue onto ZP13 and ZP14.

The effect of adsorbent dosage (g/L) on the adsorption capacities for MB onto the ZP13 and ZP14 samples is shown in Figure 5a. As the adsorbent dosage increased, the removal percentage also increased, with an increase in adsorbent dosage from 0.5 to 1.8 g/L accompanied by an increase in R% from 10–30 and from 30–95% for ZP13 and ZP14, respectively. The previous sharp increase in adsorption with adsorbent dosage is related to the increase in the ratio of active sites of the solid adsorbent/the number of MB molecules. Above 1.8 g/L, the increase in adsorbent dosage was accompanied by nearly constant R% values due to the decrease in the number of MB molecules relative to the active sites of the solid materials [38]. These results indicate that 1.8 g/L can be considered the optimal adsorption dosage value.

#### 3.2.2. Effect of Initial pH of Methylene Blue Solution

The acidity of the adsorption medium plays an important role in adsorption from solution onto the surfaces of solid materials, especially the pH effect on the ionization of adsorbate molecules and the charge present on the surface of the solid adsorbent. The effect of pH (1–12) on the adsorption of MB was studied using ZP13 and ZP14, as two selected solid adsorbent samples, with 1.8 g/L as the adsorbent dosage, for 50 mL of 200 mg/L MB solution after 24 h of shaking time, at 25 °C (Figure 5b). The lower removal percentage of MB at lower pH values (<pH_PZC_) is related to the competition between MB and H_3_O^+^ on active sites, where the latter is more easily adsorbed than MB molecules. Increasing the pH value from 1 to 8 was accompanied by a sharp increase in the removal percentage for MB onto ZP13 and ZP14, by about five and three times, respectively. At pH > 8 (pH > pH_PZC_), the surfaces of ZP13 and ZP14 accept negative charge, which is responsible for increasing the repulsion between MB ions and the negatively charged solid surface [39]. A value of pH8 was selected as the optimum pH value for adsorption of methylene blue onto the prepared solid adsorbents.

#### 3.2.3. Effect of Contact Shaking Time and Kinetic Studies

**Figure 6:**
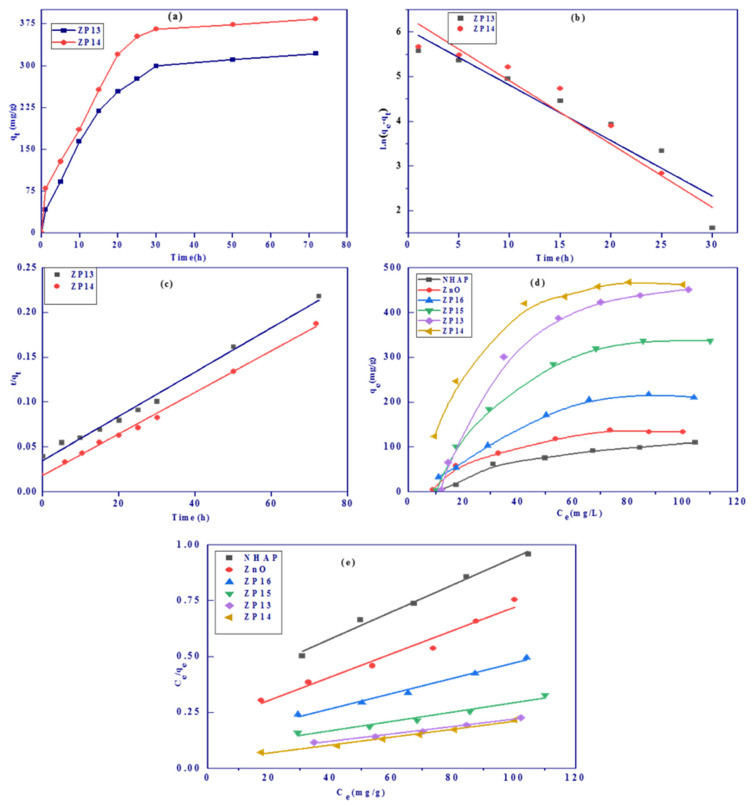
Effect of shaking time (**a**) and PFO (**b**) and PSO (**c**) kinetic models for adsorption of MB onto ZP13 and ZP14. Adsorption isotherms (**d**) and linear Langmuir plots (**e**) for adsorption of MB onto all the prepared solid adsorbents.

The relations between shaking times and adsorption capacity (*q_t_*, mg/g) for ZP13 and ZP14 samples are shown in Figure 6a. The adsorption equilibrium was achieved after about 24 h for the two investigated solid samples. At the initial stage of adsorption, the rate of adsorption was high due to the availability of free active sites that become saturated over time. The rate of adsorption slowed down after ~24 h, which may be related to the saturation of active sites on the solid adsorbent [40].

Linear kinetic model plots for pseudo-first-order (PFO) and pseudo-second-order (PSO) kinetics are shown in Figure 6b,c, respectively, and the calculated kinetic parameters are listed in Table 1. The adsorption of MB was well fitted by the pseudo-second-order equation with correlation coefficients of 0.988964 and 0.996050 for ZP13 and ZP14, respectively. The experimental adsorption capacities (*q_exp_*) calculated by the PSO model were very close to those calculated from the Langmuir adsorption model (*q_m_*, mg/g), with differences of 2.1% and 5.4% for ZP13 and ZP14, respectively. The application of the PFO kinetic model resulted in small correlation coefficients (<0.873683) and large differences between experimental adsorption capacities and the Langmuir adsorption capacities for ZP13 and ZP14. These data confirm that the adsorption of MB onto selected solid samples (ZP13 and ZP14) followed a PSO kinetic model.

#### 3.2.4. Effect of Initial Methylene Blue Concentration

Adsorption isotherms describe the relation between the equilibrium concentration (*C_e_*, mg/L) and the adsorption capacity (*q_e_*, mg/g) and are used to investigate the adsorption feasibility. Figure 6d shows the adsorption isotherms of MB onto NHAP, ZnO, ZP13, ZP14, ZP15, and ZP16 at pH 7, with 1.8 g/L as the adsorbent dosage, after 24 h, at 25 °C, with different MB concentrations (20–800 mg/L). The Langmuir adsorption isotherm model, which is based on adsorption onto a homogeneous surface without interaction between adsorbate molecules, was applied to evaluate the maximum adsorption capacity and the dimensionless separation factor for all samples. Linear Langmuir plots are presented in Figure 6e and the calculated parameters are shown in Table 2. Upon analysis of the data in Table 2, the following results were found. (i) The Langmuir adsorption model is highly applicable based on high R^2^ values (0.967441–0.996475). (ii) The maximum adsorption capacities for all the investigated solid adsorbents were found to be 134.5, 202.3, 504.9, 596.1, 428.5, and 312.4 mg/g for NHAP, ZnO, ZP13, ZP14, ZP15, and ZP16, respectively, predicting that ZP14 would exhibit the maximum adsorption capacity. (iii) NHAP showed smaller adsorbed amounts of MB than ZnO, while the formed composites had higher adsorption values than the single-component solid materials (NHAP or ZnO). The adsorption capacities increased from ZP13 to ZP14 and started to dramatically decrease from ZP15 to ZP16, with a further increase in the percentage of NHAP in the produced composites. (iv) The dimensionless separation factor (*R_L_*) ranged between 0.10977 and 0.26466, indicating the favorable adsorption of MB onto all solid adsorbents [41].

### 3.3. Photocatalytic Degradation of Methylene Blue

#### 3.3.1. Effect of Photocatalyst Dosage

**Figure 7:**
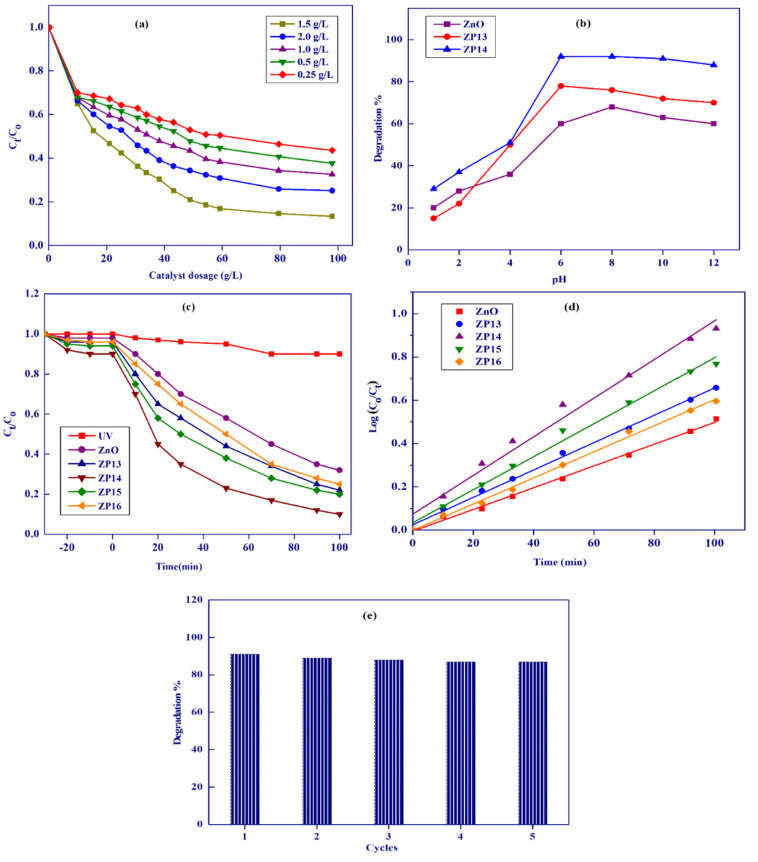
Effect of ZP14 dosage (**a**), effect of pH on activity of ZnO, ZP13, and ZP14 (**b**), photolysis and photocatalytic activity of ZnO, ZP13, ZP14, ZP15, and ZP16 (**c**), kinetics studies of ZP13 and ZP14 (**d**), and ZP14 reusability (**e**) for degradation of MB.

The effect of the photocatalyst dosage on MB degradation for ZP14 was investigated as shown in Figure 7a. After 100 min of photocatalysis, the degradation efficiencies for MB were found to be 57, 62, 67, 88, and 75% using 0.25, 0.5, 1.0, 1.5, and 2.0 g/L of catalyst, respectively, indicating that 1.5 g/L is the most efficient catalyst dosage. This observation reveals that increasing the adsorbent dosage from 0.25–1.50 g/L was accompanied by an increase in degradation efficiency due to the enhancement of light absorption, which is related to an increase in active sites on the surface of ZP14 [39]. A further increase in the catalyst dosage from 1.5 to 2.0 g/L was accompanied by a decrease in the catalyst activity by about 13%, which may be related to the turbidity effect of ZP14 particles, reducing light penetration to the surface of the catalyst and diminishing the degradation of MB [42]. A catalyst dosage of 1.5 g/L was chosen for the subsequent experiments, as the optimum value.

#### 3.3.2. Effect of Initial Solution pH on Degradation of Dye

The effect of pH on the photocatalytic degradation of MB was investigated using ZnO, ZP13, and ZP14 in the pH range of 1–12, as presented in Figure 7b. It was observed that the maximum degradation percentage of MB using the investigated solid nanocatalysts occurred at nearly pH 8. At lower pH values, the lower degradation efficiencies are related to the lower adsorption of MB, which is accompanied by lower degradation of the dye [43]. The photocatalytic activity slightly decreased above pH 8 due to the decrease in MB adsorption [44,45]. Based on the previous results, we concluded that pH 8 is the most suitable medium for photocatalytic degradation of MB onto the surface of the prepared photocatalyst.

#### 3.3.3. Comparison of Catalytic Activities of the Prepared Solid Photocatalysts

The photolysis and photocatalytic degradation of MB in the presence of ZnO, ZP13, ZP14, ZP15, and ZP16 were studied in the presence of UV radiation, as shown in Figure 7c. The activities of the investigated photocatalysts were found to be 10, 69, 78, 91, 80, and 75%, respectively, after 100 min. The degradation of MB in the absence of any photocatalyst was very low (10%), which may be related to the stability of the MB molecules. In the presence of nano zinc oxide as the photocatalyst, the degradation of MB increased to 69% due to the effect of the photocatalyst, which is responsible for the creation of a strong oxidant (OH) generated from the electron–hole pairs. The degradation efficiencies of the composites were found to be greater than those recorded for single ZnO, which may be related to the well-distributed zinc oxide nanoparticles on the matrix of NHAP [46]. The photocatalytic activities were in the order ZP14 > ZP15 > ZP16, which can be related to the content of nano zinc oxide photocatalyst in the formed composites. Zinc oxide nanoparticles, as semiconductors, are characterized by a 3.3 eV band gap at 27 °C and 60 mV binding energy, which enhances their application as a photocatalyst in the degradation of some organic pollutants [47]. Nano zinc oxide separation and reusability from the application medium after photocatalytic processes is not recommended due to the loss of fine nanoparticles and the higher particle coagulation, which is accompanied by a loss in activity. These drawbacks for nano zinc oxide applications were solved by the formation of nano zinc oxide composites with mechanically stable organic or inorganic polymers such as chitosan, graphene oxide, kaolinite, etc. [48,49,50]. After light is absorbed by the nano zinc oxide, electron–hole pairs are created between the valence and the conduction bands (Equation (11)), which are responsible for the photocatalytic degradation of methylene blue dye into volatile gases [51,52]. Both the holes and electrons take part in the oxidation process via the production of active ^•^OH, which is responsible for MB oxidation. Positive holes react with either H_2_O or –OH giving free hydroxyl radicals (Equations (12) and (13)) while electrons react with adsorbed oxygen on the surface of the catalyst, forming **^•^**OH (Equations (14)–(18)) [43].

ZnO + hυ → ZnO (e^−^ + h^+^) (11)

^−^OH + h^+^ → ^•^OH (12)

H_2_O + h^+^ → ^•^OH + H^+^ (13)

O_2_ + e^−^ → O_2_^• −^ (14)

2O_2_^• −^ + H^+^ → ^•^O_2_H + O_2_^• −^ (15)

2^•^O_2_H → H_2_O_2_ + O_2_ (16)

H_2_O_2_ + O_2_^• −^ → ^•^OH + ^−^OH + O_2_ (17)

H_2_O_2_ + e^−^ → ^•^OH + ^−^OH (18)

#### 3.3.4. Kinetic Studies for Photocatalytic Degradation of Methylene Blue

The plot of *log*(*C_o_/C_t_*) against time (*t*, min) for the Langmuir–Hinshelwood first-order kinetic model is shown in Figure 7d. The higher calculated correlation coefficients (0.96456–0.99875) confirm the suitability of the application of the Langmuir–Hinshelwood first-order kinetic model. The measured apparent first-order rate constants for ZnO, ZP13, ZP14, ZP15, and ZP16 were 0.01151, 0.01455, 0.02179, 0.01538, and 0.01403 min^−1^, respectively. The apparent rate constants increased with the increase in photocatalytic activity of the photocatalyst, and ZP14 had the maximum rate constant.

#### 3.3.5. Photocatalyst Reusability

Catalyst reusability was investigated after five cycles of application using ZP14 as the selected solid photocatalyst. After five cycles of ZP14 application, we observed that the catalyst activity decreased by only about 4% (Figure 7e), which may be related to the decrease in surface area due to particle coagulation, the reduction in the external surface area, and the loss of some surface active sites, as reported by many authors [53,54,55,56].

### 3.4. Comparison between ZP14 and Other Solid Adsorbents and Photocatalysts

A comparison of the maximum adsorption capacities and photocatalytic activities for ZP14 and other published nanomaterials is presented in Table 3 [6,46,57,58,59,60,61]. Based on the tabulated data for adsorption and photocatalytic degradation of methylene blue, we can conclude that nano zinc oxide/nanohydroxyapatite composite particles (ZP14) are promising solid nanomaterials in environmental applications. 

## 4. Conclusions

Different nanoparticles (ZnO and NHAP) and their composites in different ratios (ZP13, ZP14, ZP15, and ZP16) were prepared based on green synthesis procedures using date palm pits extract (DPPE) and eggshell precursors. The prepared extract and solid nanomaterials were investigated using various physicochemical tools, confirming the nanostructure of the prepared solid samples. Different chemical compounds such as 9-octadecenoic acid, (E), TMS derivative, palmitic acid, TMS derivative, trans-13-octadecenoic acid, and dodecanoic acid trimethylsilyl ester (23.81, 16.12, 8.72, and 8.46%, respectively) were detected in DPPE, which acted as an anticoagulant for the prepared particles. Prepared nanomaterials had particle sizes in the range of 42.3–66.1 nm, and pH_PZC_ ranged between 7.4 and 8.4, according to the presence of different chemical functional groups. 

Comparing the adsorption and photocatalytic degradation of methylene blue dye in the presence of the prepared solid nanomaterials, we concluded that, at higher concentrations of MB (>20 mg/L), the prepared solid nanocomposite was effective in the adsorption process, with recorded adsorption capacities of 596.1 mg/g, but required a longer shaking time (24 h). The prepared nanocomposite exhibited fast and effective photocatalytic degradation of MB by about 91% in a very short time (<100 min) but at a lower concentration of dye (≤20 mg/L). These results indicate that ZnO/nanohydroxyapatite composites are promising nanomaterials for the adsorption and photocatalytic degradation of dyes. The unique properties of the ZnO/nanohydroxyapatite composite prepared using green synthesis methods encourage us to investigate its use in other medical and environmental applications. 

## Figures and Tables

**Table 1:** Pseudo-first-order and pseudo-second-order kinetic model parameters for MB adsorption onto ZP13 and ZP14.

Sample	q_m_ (mg/g)	PFO Kinetic Model			PSO Kinetic Model		
		q_exp_ (mg/g)	k_1_ (h^−1^)	R^2^	q_exp_ (mg/g)	k_2_ (g/mg/h)	R^2^
ZP13	504.9	414.1	0.1204	0.873683	494.8	1.78 × 10^−4^	0.988964
ZP14	596.1	501.2	0.1421	0.863212	561.0	3.04 × 10^−4^	0.996050

**Table 2:** Langmuir adsorption parameters for MB adsorption onto prepared solid adsorbents.

Parameter	NHAP	ZnO	ZP13	ZP14	ZP15	ZP16
q_m_ (mg/g)	134.5	202.3	504.9	596.1	428.5	312.4
b (L/mg)	0.01819	0.02610	0.05479	0.03061	0.02510	0.02651
R_L_	0.26466	0.16077	0.14045	0.10977	0.16611	0.15873
R^2^	0.985361	0.996475	0.983010	0.989900	0.967441	0.983482

**Table 3:** Photocatalytic degradation and adsorption of MB using ZP14, compared with other solid materials.

Solid Nanomaterials	Maximum Capacity		Reference
	Adsorption	Photocatalysis	
Cu/nanogoethite	12.8 mg/g	NA	[57]
Chitosan/montmorillonite composite	181 mg/g	NA	[6]
Fe_3_O_4_/activated montmorillonite nanocomposite	106.4 mg/g	NA	[58]
Fe-AC	191.0 mg/g	NA	[46]
In/ZnO nanoparticles	NA	89%	[59]
SnO_2_ nanoparticles	NA	90%	[60]
Barium monoferrite	NA	90%	[61]
ZP14	596.1 mg/g	91%	[This study]

## Data Availability

Data on the compounds are available from the author.

## Supplementary Materials

The following are available online at https://www.mdpi.com/article/10.3390/nano12010049/s1. Figure S1: EDX elemental microanalysis of NHAP, ZnO, and composite samples; Table S1: GC-MS differentiation of the compounds detected in the date palm pits extract.
