# Supplementary material for: Green Synthesis of Nano Zinc Oxide/Nanohydroxyapatite Composites Using Date Palm Pits Extract and Eggshells: Adsorption and Photocatalytic Degradation of Methylene Blue
Source: Nanomaterials (Basel). 2021 Dec 24;12(1):49. doi: 10.3390/nano12010049 (PMC8746312; doi:10.3390/nano12010049)
Supplement: Supplementary file 1 [file nanomaterials-12-00049-s001.zip › nanomaterials-1489438-supplementary.pdf]

Supplementary materials

Green Synthesis of Nano Zinc Oxide/Nanohydroxyapatite Composites Using Date Palm Pits Extract and Eggshells: Adsorption and Photocatalytic Degradation of Methylene Blue

Maha S. Elsayed [1,*], Inas A. Ahmed [2], Dina M. D. Bader [2] and Asaad F. Hassan [3]

[1] Central Laboratory of Date Palm Research and Development, Agricultural Research Center, Giza 12619, Egypt
[2] Department of Chemistry, Faculty of Science, King Khalid University, Abha 62224, Saudi Arabia; eaahmed@kku.edu.sa (I.A.A.); ddeyaa@kku.edu.sa (D.M.D.B.)
[3] Chemistry Department, Faculty of Science, Damanhour University, Damanhour 22511, Egypt; asmz68@sci.dmu.edu.eg
* Correspondence: mahasobhy1000@yahoo.com; Tel.: +20-106-257-8855

Table S1. GC-MS differentiation of the compounds detected in the date palm seeds extract

Peak	RT	Name	Formula	Area	Area Sum %
1	13.337	Octadecane, 6-methyl-	$C_{19}H_{40}$	3113268.1	0.64
2	13.767	Decane, 2,3,5,8-tetramethyl-	$C_{14}H_{30}$	4090752.6	0.83
3	14.083	Butylated Hydroxytoluene	$C_{15}H_{24}O$	13840947	2.82
4	14.43	Benzoic acid, 3,5-bis(1,1-dimethylethyl)-4-hydroxy-, ethyl ester	$C_{17}H_{26}O_3$	7696635	1.57
5	15.575	Dodecanoic acid, trimethylsilyl ester	$C_{15}H_{32}O_2Si$	41436959	8.46
6	16.185	Tetradecane, 2,6,10-trimethyl-	$C_{17}H_{36}$	3266406.7	0.67
7	17.384	9-Octadecenoic acid (Z)-	$C_{18}H_{34}O_2$	6094113.9	1.24
8	17.625	Myristic acid, TMS derivative	$C_{17}H_{36}O_2Si$	31837903	6.5
9	18.363	Tetradecane, 2,6,10-trimethyl-	$C_{17}H_{36}$	5402844.2	1.1
10	19.516	Palmitic Acid, TMS derivative	$C_{19}H_{40}O_2Si$	79002808	16.12
11	21.001	9,12-Octadecadienoic acid (Z,Z)-, TMS derivative	$C_{21}H_{40}O_2Si$	26696317	5.45
12	21.046	9-Octadecenoic acid, (E)-, TMS derivative	$C_{21}H_{42}O_2Si$	116682823	23.81
13	21.754	9,12,15-Octadecatrienoic acid, 2-(acetyloxy)-1-[(acetyloxy)methyl]ethyl ester, (	$C_{25}H_{40}O_6$	3102104.6	0.63
14	22.553	9,12,15-Octadecatrienoic acid, 2-[(trimethylsilyl)oxy]-1-[[(trimethylsilyl)oxy]m	$C_{27}H_{52}O_4Si_2$	5160684.2	1.05
15	23.728	Di-n-octyl phthalate	$C_{24}H_{38}O_4$	17280429	3.53
16	24.022	1-Monopalmitin, 2TMS derivative	$C_{25}H_{54}O_4Si_2$	9580733.8	1.96
17	25.243	1-Monooleoylglycerol, 2TMS derivative	$C_{27}H_{56}O_4Si_2$	14977367	3.06
18	27.383	13-Heptadecyn-1-ol	$C_{17}H_{32}O$	2030498.9	0.41
19	27.873	E-9-Tetradecenoic acid	$C_{14}H_{26}O_2$	4554852.5	0.93
20	28.174	Dodecanoic acid, 3-hydroxy-	$C_{12}H_{24}O_3$	10979353	2.24
21	30.706	Ergosta-5,22-dien-3-ol, acetate, (3.beta.,22E)-	$C_{30}H_{48}O_2$	3338742.6	0.68
22	36.267	9,12-Octadecadienoyl chloride, (Z,Z)-	$C_{18}H_{31}ClO$	21144347	4.32
23	37.187	trans-13-Octadecenoic acid	$C_{18}H_{34}O_2$	42737800	8.72
24	43.117	8,11,14-Eicosatrienoic acid, (Z,Z,Z)-	$C_{20}H_{34}O_2$	15966377	3.26

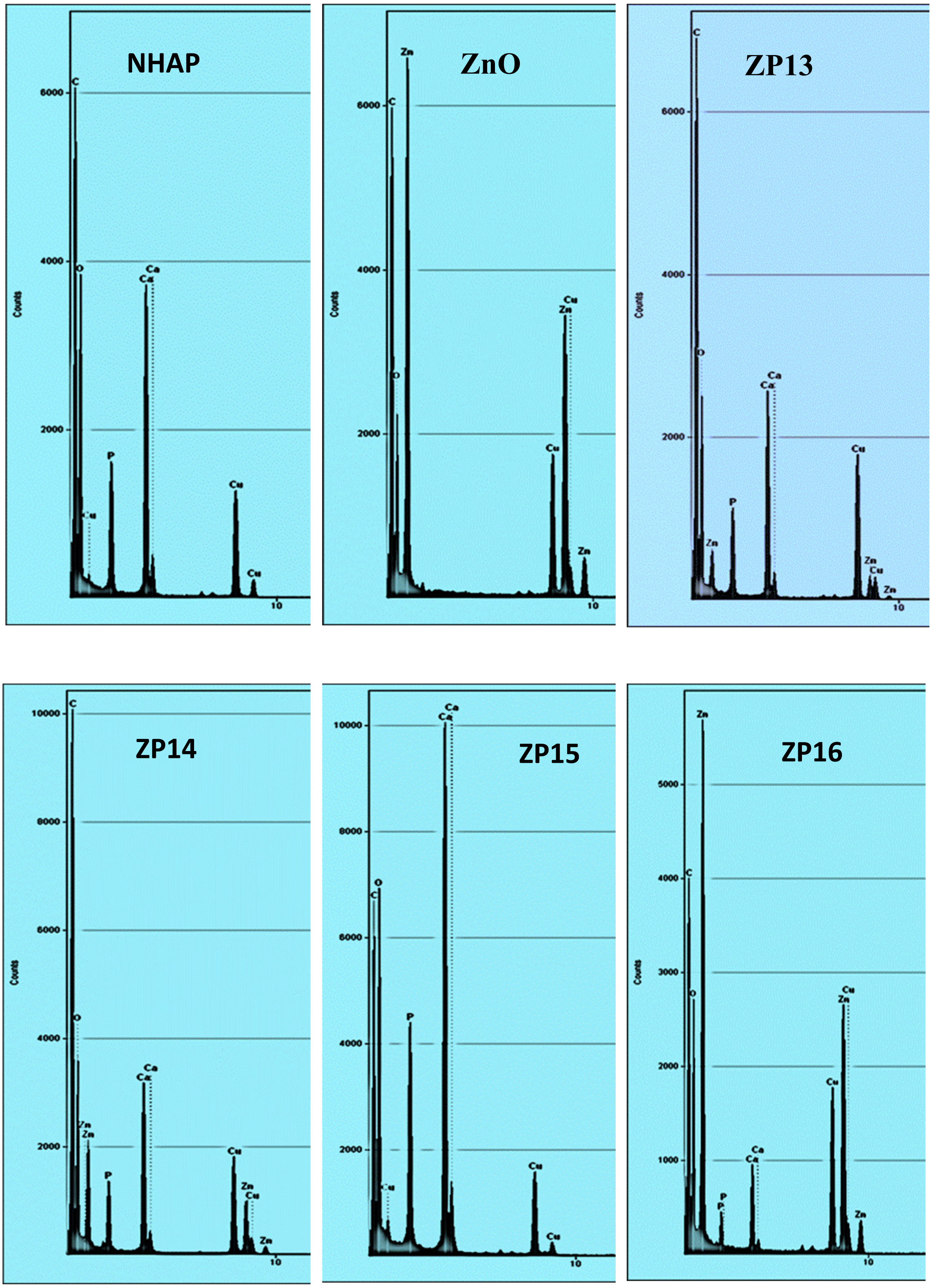

Figure S1. EDX elemental microanalysis of NHAP, ZnO and composite nano samples
